# Determinants of modern contraceptive use among sexually active men in Ethiopia; using EDHS 2016 national survey

**DOI:** 10.1186/s40834-020-00108-7

**Published:** 2020-05-06

**Authors:** Tamrat Shaweno, Zerihun Kura

**Affiliations:** 1 Department of Epidemiology, Faculty of Public Health, Jimma University, Jimma, Ethiopia; 2 Biostatistics Unit, Department of Epidemiology, Faculty of Public Health, Jimma University, Jimma, Ethiopia

**Keywords:** Modern contraceptive, Male method, EDHS 2016, Ethiopia

## Abstract

**Background:**

Recently, the focus of family planning programs has changed from female oriented to men oriented, or both partner oriented to have effective outcomes. Although, contraceptive use among sexually active women was exhaustively researched; there is still a huge gap on modern contraceptive use and its determinants among sexually active men in Ethiopia.

**Objectives:**

We assessed the determinants of contraceptive use among sexually active men in Ethiopia using national survey data.

**Method:**

The data source for this study was the 2016’s Ethiopian Demographic and Health Survey of men aged above 15–59 years. We analyzed data of 12, 688 sexually active men in the past 12 months prior to the survey using STATA version 14.1. Bivariate and multivariable multinomial logistic regression analysis was conducted and statistical significance was set at *p* value < 0.05.

**Result:**

From a total of 12, 688 sexually active men in Ethiopia, 9378 (73.9%) didn’t use any modern contraception or use traditional methods, 2394 (18.9%) use partner methods for those who reported using a method through their partner (such as pill, IUD, injections, female sterilization and Norplant) and the rest 916 (7.2%) used male methods for those who reported using male only methods (such as condoms and male sterilization). In the adjusted multinomial logistic regression model, men’s age categories 25–34 years (AOR:2.0; 95%CI = 1.5–2.5), 35–44 (AOR: 2.8; 95%CI = (2.0–3.8), and 45^+^ years (AOR: 1.5; 95%CI = 1.0–2.6), being rural resident (AOR: 1.60; (95%CI = 1.3–2.2), married and living with partner (AOR: 0.03; (95%CI = 0.01–0.06), who attended secondary (AOR:1.2; (95%CI = 0.8–1.9) and higher (AOR: 1.4; (95%CI = 1.2–2.5) education, whose partner was working (AOR: 1.6; (95%CI = 1.3–2.2), having three and above children (AOR: 0.5; (95%CI = 0.3–0.8), reading newspaper/magazines at least once a week or less than once a week, listening to radio at least once a week, watching television at least once a week and watching television less than once a week were significantly associated with use of male method than traditional/no method as compared to their respective references.

**Conclusion:**

Modern contraceptive use among sexually active men in Ethiopia is low and multiple factors determined it. Close monitoring and supporting of sexually active men with age above 25 years, rural background, higher educational level, whose partner was working, having three and more children and little exposure to media may increase men’s use of modern contraceptives.

## Introduction

The importance of modern contraceptive use is now well recognized, not only to improve women and child health, but also to contribute to related issues such as gender equality, better child health, an improved response to HIV, greater education outcomes and poverty reduction [1]. To reaffirm the promise of universal access to reproductive health and family planning, the international community is giving special attention to regions and countries lagging behind [1–3]. Huge gaps exist in demand for family planning satisfied with modern contraceptives in countries where overall contraceptive use is low or where many sexually active groups rely on traditional methods of contraception [2, 3]. At the end of 2015, less than half of total demand for contraceptives was being met with modern methods in 54 countries (of which more than 60% are in Africa) [4]. Between 2015 and 2030, the time period of the 2030 Agenda for Sustainable Development, contraceptive use is projected to increase particularly in countries where less than half of reproductive age groups currently use contraception [4]. In line with this vision, Ethiopia has committed to increase the modern contraceptive prevalence rate from 35% to 55% amongst married women by 2020 and reducing the total fertility rate from 4.6 to 3.0 [5]. Family planning programs have traditionally focused primarily on women; men being over looked and given lesser attention [6]. Although, some studies have highlighted the influence of men on reproductive decisions such as contraceptive use, still information is scarce with regard to contraceptive use among sexually active men [6, 7]. Men, particularly in male-controlled contexts, play a great role in influencing fertility choices [8]. However, male involvement in family planning remains limited [9, 10]. In addition, research examining determinants of couples’ modern contraceptive use has ignored men’s crucial role [11, 12], which has eventually resulted in underlining the idea of contraceptive use as women’s concern, leaving little or no role for men [13–15]. It was observed [16] that men, particularly in low socio economic settings, prefer large numbers of children both as a source of labor and profitable gain, and as a source of respect. Men’s role as central decision makers, are believed to determine men’s and couples’ use of contraceptives [17, 18]. A men’s agreement of the use of contraception is a key for effective family planning programs [19, 20]. A study illustrated that men had complete decision making supremacy and the ability to result in compliance or submission from their partners [16].

Even though, different family planning programs excluded the participation of men [20, 21], in recent years, efforts are ongoing to extend men’s participation in reproductive health and use of contraceptives [22]. Findings indicated that discussion of family planning with a health worker, region, education, wealth index, number of surviving children and fertility preference were most significantly associated with modern contraceptive use among men [23, 24]. Studies from different parts of Ethiopia indicated that, over 90% of men supported and approved of using family planning; however, 36% of men didn’t know about male contraceptive methods [25, 26]. But, to advocate including men in contraceptive programs, information is still scarce on determinants of modern contraceptive use among men in Ethiopia [23, 24]. Moreover, the variations of modern contraceptive use among sexually active men in Ethiopia by different variables including socio-economic characteristics, interaction with health system and access to media, and behavioral and attitudinal factors remains limited. We hypothesized that, socio-demographic factors, interaction with health system, access to media and behavioral/attitudinal factors influence modern contraceptive use among sexually active men in Ethiopia. A better understanding of determinants of modern contraceptive use among sexually active men in Ethiopia could be helpful to develop intervention strategies relevant to Ethiopia. Therefore, this study aimed to explore determinant factors that influence modern contraceptive use among sexually active men in Ethiopia using 2016 EDHS national survey data.

## Methods

### Study design and data set

The data set used for this study was obtained from 2016 Ethiopian Demographic Health Surveys conducted from January 18, 2016 to June 27, 2016, across the country. The survey was a population-based cross-sectional study and it is available from the MEASURE DHS database at https://dhsprogram.com/data/available-datasets.cfm. For the surveys, there were two stages. In the first stage, the total of 645 clusters (202 in urban and 443 in rural) were randomly selected proportional to the household size from the sampling strata and in the second stage, 28 households per cluster were selected using systematic random sampling. The sample allocations were derived with information obtained from the 2011 EDHS. From a total of 16, 533 men, 14, 176 were eligible for an interview. A total of 12,688 eligible men were interviewed yielding a response rate of 89.5% [27]. Data were weighted to adjust for differences in probability of selection and to adjust for non-response. Our analysis was restricted to the 12, 688 (weighted) men who reported being sexually active in the 12 months prior to the survey and we excluded men who reported that either them or their partners were infecund.

### Study variables

The outcome variable for this study was modern contraceptive use. We categorized modern contraceptive use into three outcome categories. These included: ‘traditional/no method’ for those who reported current use of traditional or natural methods (like periodic abstinence, lactational amenorrhea and use of withdrawal method) or not using modern contraceptive methods which are ineffective in pregnancy prevention; ‘partner method’ for those who reported use of a method by their partner (like injections, pills, IUD, norplant, female sterilization and); and ‘male method’, for those who reported using male only methods (like male sterilization and condom use). The explanatory variables were grouped into five categories to see their influence on modern contraceptive use. These included socio-demographic variables (age, residence, number of children and marital status), socio-economic factors (wealth index, education and current working status of the partner), interaction with health system (discussed family planning with health worker), access to media (frequency of reading newspaper/magazine, frequency of listening to radio, frequency of watching TV) and behavioral/attitudinal factors (belief that women who use contraceptive become promiscuous and belief that contraception is a women’s business).

### Statistical analysis

Bivariate analyses were done to assess the association between predictor variables and outcome variable of the study using X^2^ tests. We selected multinomial logistic regression because it is the most suitable for the study of the outcome with three categories. Four multinomial regression models were fitted in this study. In model I we included socio demographic and partners’ working status while controlling for other covariates. Model II included socio-demographic variables, partners’ working status and total children ever born while controlling for the wealth index variables and variables related to media. Model III included socio demographic variables, partners’ working status, total number of children ever born and wealth index while controlling for access to media. In the final model all explanatory variables were included into the model. All analyses were weighted to account for differences in sampling probabilities. Those predictor variables significantly associated with outcome variable were included in the multivariable logistic regression model in which the odds ratio with 95% confidence intervals was estimated to identify the predictors of modern contraceptive use (male method) in Ethiopia. A *p*-value less than 0.05 were employed to declare the statistical significance.

## Result

### Characteristics of the study participants

From a total of 12, 688 sexually active men in Ethiopia, 9378 (73.9%) didn’t use any contraception or use traditional methods, 2394 (18.9%) use partner methods and the rest 916 (7.2%) did use male methods. Approximately, more than one third (35%) of the sexually active men were in the age group 15–24 years and more than two third (69.5%) were from the rural community. Wirth regard to marital status, more than half (56.8%) of men were married and living in marital union. Only few 1607 (12.7%) of the respondents achieved higher education whereas, majority of the respondents attended primary education. Slightly, four men out of five (83.5%) were currently working and majority have no child at the time of the survey (46.3%). With regard to interaction with the health system, exactly three fourth of the respondents didn’t discuss about contraception with the health care provider. Only, about 33.9% were categorized as richest in economic class whereas, the rest 15.2, 13.7, 14.3 and 22.9% were richer, middle, poorer and poorest, respectively. With regard to behavioral factors, more than four-fifth disagree that women who use contraceptive become promiscuous and contraception is a women’s business [Table 1].

**Table 1:** Sample characteristics of sexually active men 15–59 years in Ethiopia (2016 EDHS) (weighted)

Characteristics	Number (N)	Percent (%)
**Contraceptive use**		
None/Traditional methods	9378	73.9
vPartner methods	2394	18.9
Male methods	916	7.2
**Socio-demographic factors**		
**Age**		
45^+^	1979	15.6%
35–44	2592	20.4%
25–34	3615	28.5%
15–24	4502	35.5%
**Residence**		
Urban	3866	30.5%
Rural	8822	69.5%
**Marital status**		
Ever married but not together	375	3.0%
Married and living with partner	7208	56.8%
Never union	5105	40.2%
**Education level of respondents**		
Higher	1607	12.7%
Secondary	2233	17.6%
Primary	5331	42.0%
no education	3517	27.7%
**Current working**		
Yes	10,595	83.5%
No	2093	16.5%
**Total children over born**		
3 and above	4689	37.0%
1–2 children	2126	16.8%
no child	5873	46.3%
**Socio-economics factors**		
**Wealth index**		
Poorest	2908	22.9%
Poorer	1810	14.3%
Middle	1739	13.7%
Richer	1925	15.2%
Richest	4306	33.9%
**Interaction with health system**		
**Discussed FP with health worker**		
Yes	3159	24.9%
No	9529	75.1%
**Access to media**		
Frequency of reading newspaper/magazine		
At least once a week	1350	10.6%
less than once a week	2466	19.4%
not at all	8872	69.9%
**Frequency of listening to radio**		
At least once a week	3793	29.9%
less than once a week	3046	24.0%
not at all	5849	46.1%
**Frequency of watching TV**		
At least once a week	3433	27.1%
less than once a week	3040	24.0%
not at all	6215	49.0%
**Behavioral/attitudinal factors**		
**Women who use contraceptive become promiscuous**		
Don’t know	796	6.3%
Agree	1262	9.9%
Disagree	10,630	83.8%
**Number of wives/partners**		
Two and more wives	472	3.7%
one wife	6736	53.1%
No wife	5480	43.2%
Contraception is a women’s business		
Don’t know	690	5.4%
Agree	1145	9.0%
Disagree	10,853	85.5%

*TV Television

### Prevalence of modern contraceptive use among sexually active men

The prevalence of modern contraceptive use in relation to selected socio-demographic characteristics, behavioral factors and access to media was illustrated in Table 2. Concerning the socio-economic characteristics, a significant positive association was observed between age and use of contraceptive partner method. Sexually active men aged between 25 and 34 years (COR:1.6; 95%CI = 1.4–1.9), *P < 0.001*)), 35–44 years (COR:0.7; 95%CI = (0.6–0.9), *P < 0.001*)), and aged above 45 years (COR:0.3; 95%CI = (0.2–0.4), *p < 0.001*)), were more likely to use a partner method compared to none /traditional method users, respectively. On the contrary, men above 35 years (COR:5.9; 95%CI = 5.1–6.8), *P < 0.001*)), were less likely to use a male method than traditional or no method, compared to those men in the age group 15–24 years. Men with urban residence were more likely to use both a partner method (COR:1.5; 95%CI = (1.3–1.6), *P = 0.007*)), and male method (COR:7.5; 95%CI = (6.4–8.7), *P < 0.001*)) compared to those men with rural residence. Married men either living (COR:7.8; 95%CI = (6.- 9.0), *P < 0.001*)) or not living with the partner (COR:7.8; 95%CI = (5.9–10.3), *P < 0.001*)) were more than seven times to report use of a partner method and married men living with the partner (COR:0.1; 95%CI = (0.08–0.12), *P < 0.001*)) were less likely to use a male method compared to traditional method users or non-users. Men with primary (COR:3.8; 95%CI = (2.8–5.3), *P < 0.001*)), secondary (COR:11.7; 95%CI = (8.5–16.2), *P < 0.001*)), and; higher and above (COR: 23.2; 95%CI = (16.8–32.0), *P < 0.001*)) educational status were more likely to use male method than traditional method or non-use of any method compared to those with no registered educational status. Similarly, men with the same educational status were more likely to use partner method too, but the odds of use of male method are significantly higher compared to partner method. Men having one or two child (COR:6.6; 95%CI = (5.8–7.5), *P < 0.001*)); and three or more children (COR:3.5; 95%CI = (3.1–3.9), *P < 0.001*)) were more likely to use partner method but less likely to use a male method compared to those men with no children. Men from economic classes of richer (COR: 0.2; 95%CI = (16.8–32.0), *P < 0.001*)), middle (COR: 0.1; 95%CI = (0.1–0.2), *P < 0.001*), poorer (COR: 0.1; 95%CI = (0.1–0.2), *P < 0.001*), and poorest (COR: 0.1; 95%CI = (0.1–0.2), *P < 0.001*) were less likely to use male method as compared richest men. Men who discussed FP (family planning) with health worker (COR: 2.4; 95%CI = (2.2–2.7), *P < 0.001*) were more than two times to use a partner method compared to use traditional methods/no methods. Conversely, these men were less likely to use a male method than traditional or no use of any method compared to those who didn’t discuss about FP with health worker (COR: 0.8; 95%CI = (0.7–1.0), *P < 0.001*). Men who agreed that, women who use contraceptive becomes promiscuous were less likely to use a partner method (COR: 0.8; 95%CI = (0.7–0.9)), *P < 0.002*) or a male method (COR: 0.7; 95%CI = (0.6–0.9), *P < 0.016*) than use of traditional method /no method as compared to men who disagreed to the thought that, women who use contraceptive become promiscuous. (For details see Table 2).

**Table 2:** Bivariate association between modern contraceptive use and various background characteristics, EDHS 2016, Ethiopia (weighted)

Characteristics	Partner method vs. none/traditional method			Male method vs. none/traditional method		
Age respondents	COR	*P*-value	%CI	COR	*P*-value	%CI
45^+^	3.7	0.001	3.2–4.4	0.3	0.001	0.2–0.4
35–44	5.9	0.001	5.1–6.8	0.7	0.001	0.6–0.9
25–34	5.3	0.001	4.6-, 6.1	1.6	0.001	1.4–1.9
15–24	0^b^			0^b^		
**Residence**						
Urban	1.5	0.077	1.3–1.6	7.5	0.001	6.4–8.7
Rural	0^b^			0^b^		
**Marital status**						
Ever married but not together	7.8	0.001	5.9–10.3	1.7	0.001	1.3–2.3
Married and living with partner	7.8	0.001	6.7–8.9	0.1	0.001	0.1–0.2
Never union	0^b^			0^b^		
**Education of respondents**						
Higher and above	2.0	0.001	1.7–2.3	23.2	0.001	6.9–32.0
Secondary	1.0	0.639	0.9–1.2	11.7	0.001	8.5–16.2
Primary	1.0	0.824	0.9–1.1	3.8	0.001	2.8–5.3
None	0^b^			0^b^		
**Current working**						
Yes	6.0	0.001	4.8–7.4	1.3	0.007	1.1–1.5
No	0^b^			0^b^		
**Total children over born**						
3 and above	3.5	0.001	3.1–3.9	0.1	0.099	0.1, 0.2
1–2 children	6.6	0.001	5.8–7.5	0.5	0.595	0.4–0.5
No child	0^b^			0^b^		
**Socio-economics factors**						
**Wealth index**						
Poorest	0.3	0.001	0.2–0.6	0.1	0.001	0.1–0.2
Poorer	0.7	0.001	0.6, 0.8	0.1	0.001	0.1–0.2
Middle	0.9	0.164	0.8–1.0	0.1	0.001	0.1–0.2
Richer	1.0	0.687	0.9–1.1	0.2	0.001	0.2–0.3
Richest	0^b^			0^b^		
**Interaction with health system**						
**Discussed FP with health worker**						
Yes	2.4	0.001	2.2–2.7	0.8	0.031	0.7–1.0
No	0^b^			0^b^		
**Access to media**						
Frequency of reading newspaper/magazine						
At least once a week	2.0	0.001	1.7–2.3	5.1	0.001	4.3–6.2
Less than once a week	1.634	0.001	1.5–1.8	3.7	0.001	3.2–4.4
Not at all	0^b^			0^b^		
**Frequency of listening to radio**						
At least once a week	2.0	0.001	1.8–2.3	4.7	0.001	4.0–5.6
Less than once a week	1.323	0.001	1.2–1.5	2.5	0.001	2.1–3.1
Not at all	0^b^			0^b^		
**Frequency of watching TV**						
At least once a week	2.2	0.001	2.0–2.5	9.7	0.001	2.5–11.8
Less than once a week	1.9	0.001	1.7–2.1	3.6	0.001	2.1, 4.6
Not at all	0^b^			0^b^		
**Behavioral/attitudinal factors**						
**Women who use contraceptive become promiscuous**						
Don’t know	0.2	0.001	0.1–0.3	0.1	0.001	0.1–0.2
Agree	0.8	0.002	0.7–0.9	0.7	0.016	0.6–0.9
Disagree	0^b^			0^b^		
**Number of wives/partners**						
Two and more wives	3.2	0.001	2.5–4.1	0.2	0.001	0.01–0.13
One wife	6.0	0.001	5.3–6.8	0.1	0.001	0.1–0.2
No wife	0^b^			0^b^		
Contraception is a women’s business						
Don’t know	0.1	0.001	0.1–0.2	0.2	0.001	0.1–0.3
Agree	0.8	0.010	0.7–1.0	0.5	0.001	0.4–0.7
Disagree	0^b^			0^b^		

*COR adjusted odds ratio*, CI* confidence interval*, 0*^*b*^ reference category*, FP* family planning *TV* television

### Determinants of modern contraceptive use among sexually active men

In the adjusted multinomial logistic regression model, age of men, residence, marital status, partners working, number of ever born children, access to media, frequency of listening to radio and frequency of watching TV were significantly associated with use of male method than partner method compared to use of traditional method / no method. Men aged above 25 years were less likely to report use of partner method than use of traditional /no method compared to men whose age 15–24 years. Contrariwise, these men were less likely to use partner method than use of traditional/no method compared to men aged 15–24 years. Men aged 25–34 years (AOR: 2.0; 95%CI = (1.5–2.5), 35–44 years (AOR: 2.8; 95%CI = (2.0–3.8)), and 45^+^ years (AOR: 1.5; 95%CI = (1.0–2.6)) were approximately, 2.0 (*p* = 0.004), 3.0 (*p* < 0.01) and 1.5 times (*p* < 0.01) more likely to use male methods than traditional /no method respectively, compared to men aged 15–24 years. Men with urban residence were nearly two times more likely to use male method than traditional/no methods compared to men from rural residence (AOR: 1.60; (95%CI = 1.3–2.2), (*p* < 0.001)). Men who were ever married and living with the partner were 0.03 times, (*p* < 0.001) less likely to use male method than traditional/no method compared to men not in marital union (AOR: 0.03; (95%CI = 0.01–0.06)), (*p* < 0.001)). Men whose educational status was secondary (AOR: 1.2; (95%CI = 0.8–1.9), (*p* < 0.024)) and higher (AOR: 1.4; (95%CI = 1.2–2.5), (*p* < 0.001)) were significantly associated with use of male method than traditional /no method compared to men who didn’t attend formal education. There were no significant associations observed among partner method users of similar educational category. Men whose partners’ work was significantly associated with use of male method (AOR: 1.6; (95%CI = 1.3–2.2), (*p* < 0.001)) than traditional /no method as compared to men whose partners were not working. Having one or two child was not significantly associated with use male method (AOR: 1.2; (95%CI = 0.8–1.8), (*p* > 0.050)) than traditional/no method as compared to men having no children. Men having more than three and above children were 0.5 times less likely to use male method than traditional/no method as compared to men having no children (AOR: 0.5; (95%CI = 0.3–0.8), (*p* < 0.001)).

With regard to access to media, men reading newspaper /magazine at least once a week (AOR: 1.4; (95%CI = 1.1–1.7), (*p* < 0.001)) and below once a week (AOR: 1.5; (95%CI = 1.2–1.8), (*p* < 0.001)) were more than 1.5 times more likely to use male method than traditional methods as compared to men not reading newspaper /magazine at all. Similarly, men listening to radio at least once a week were more likely to use male method (AOR: 1.5; (95%CI = 1.2–1.9), (*p* < 0.002)) than traditional /no method as compared to men not listening to radio at all. Men watching TV at least once a week (AOR: 1.9; (95%CI = 1.5–2.4), (*p* < 0.001)) and below once a week (AOR: 1.5; (95%CI = 1.1–1.9), (*p* < 0.003)) were approximately two times more likely to use male method than traditional /no method as compared to men not watching TV at all **[**Table 3**].**

**Table 3:** Multivariate association between modern contraceptive use and various background characteristics, EDHS 2016, Ethiopia (weighted)

Characteristics	Model I		Model II		Model III		Model IV	
Age respondents	aOR	%CI	aOR	%CI	aOR	%CI	aOR	%CI
45^+^	1.9	1.3–3.0	1.7	1.2–2.9	1.6	1.1–2.8	1.5	1.0–2.6
35–44	3.2	2.3–4.3	3.1	2.2–4.2	3.0	2.2–4.1	2.8	2.0–3.8
25–34	2.3	1.9–2.7	2.2	1.8–2.7	2.1	1.7–2.6	2.0	1.5–2.5
15–24	0^b^		0^b^		0^b^		0^b^	
**Residence**								
Urban	2.0	1.6–2.5	1.9	1.5–2.4	1.8	1.4–2.3	1.6	1.3–2.2
Rural	0^b^		0^b^					
**Marital status**								
Ever married but not together	1.2	0.8–1.9	1.1	0.7–1.7	1.0	0.7–1.8	0.9	0.6–1.4
Married and living with partner	0.1	0.06–0.1	0.1	0.05–0.09	0.04	0.03–0.08	0.03	0.01–0.06
Never union	0b		0b		0^b^		0^b^	
**Education level**								
Higher	2.1	1.5–3.1	1.8	1.4–3.0	1.6	1.3–2.8	1.4	1.2–2.5
Secondary	1.5	1.1–2.2	1.4	1.1–2.1	1.3	1.0–2.0	1.2	0.8–1.9
Primary	1.3	0.9–1.8	0.9	0.8–1.7	0.8	0.7–1.6	0.6	0.6–1.4
No education	0^b^		0^b^		0^b^		0^b^	
**Partners working status**								
Yes	2.0	1.6–2.5	1.8	1.5–2.4	1.7	1.4–2.3	1.6	1.3–2.2
No	0b							
**Total children ever born**								
3 and above			0.3	0.1–0.6	0.4	0.2–0.7	0.5	0.3–0.8
1–2 children			0.9	0.6–1.5	1.1	0.7–1.7	1.2	0.8–1.8
No child			0^b^		0^b^		0^b^	
**Wealth index**								
Rich					1.2	0.8–1.6	1.3	0.9–1.7
Medium					0.6	0.4–1.1	0.8	0.5–1.2
Poor					–		–	
Discussed **FP with health worker**								
Yes							0.7	0.6–0.8
No							0^b^	
**Access to media**								
Frequency of reading newspaper /magazine								
At least once a week							1.4	1.1–1.7
Less than once a week							1.5	1.2–1.8
Not at all							0^b^	
**Frequency of listening Radio**								
At least once a week							1.5	1.2–1.9
Less than once a week							1.3	0.9–1.6
Not at all							0^b^	
**Frequency of watching TV**								
At least once a week							1.9	1.5–2.4
Less than once a week							1.5	1.1–1.9
Not at all							0^b^	

*aOR adjusted odds ratio*, CI* confidence interval*, 0*^*b*^ reference category*, TV* television

## Discussion

Currently, provision of safe, effective and affordable modern contraceptive methods is emphasized to achieve high levels of demand satisfied for family planning through addressing both women’s and men’s sexual and reproductive health needs [28]. This study illustrated determinants of modern contraceptive use among sexually active men in Ethiopia using EDHS 2016 national survey. In the final model a total of eight variables showed significant association with modern contraceptive use among sexually active men. In this study, age categories of 25–34 and 35–44 were more likely to use male method or partner method than traditional/no method as compared to men in the age categories of 15–24 years’ age. Compared to studies from Kenya [29] and Tanzania [30] this finding is not consistent in which men aged 25 years and above were less likely to report use of a partner method than use of a traditional/no method compared to those under 25 years. Part of explanation for this variation could be the increased awareness of youth reproductive health in Ethiopia might have increased the uptake of male method too. With regard to residence; men residing in the urban part of the country were more likely to use male method than traditional/ no method as compared to men residing in rural part of Ethiopia. Compared to studies from Kenya [29], and Nigeria [31], this finding was consistent in which men from rural community were less likely to use male method than traditional /no method as compared to men from urban. But, it was inconsistent to a study [32] in which residents of rural community were more likely to use modern contraceptives. In this study, men married and living with their partner were less likely to use male method than traditional /no method as compared to men not in marital union at all. Although, we didn’t find related literature on this part, our explanation for this could be men living with their partner might have full confidence and trust with their partner so that they can be negligible in use of male method. Concerning to educational status, men who attended secondary and higher education were more likely to use male method than traditional /no method as compared to men who didn’t attend any education. Compared to studies from Uganda [23, 32], this finding is consistent in that men with secondary and above level of education were more likely to use modern contraceptives.

Men, whose partners were currently working, were more likely to use partner method or male method than traditional/no method as compared to men whose partners were not currently working. Compared to studies from other settings, this finding was consistent with the study from Nigeria [33] and in which men whose partners were currently working were more likely to utilize modern contraceptives.

In this study, men with number of ever born children equals three or more were less likely to use male method than traditional/no method compared to men without children. Compared to other studies [33], this finding was not consistent in that men with increasing number of children were more likely to use modern contraceptives. The difference can be explained from, men with large family size in Ethiopia may fear to have additional number of children due to economic and social problems, thus they might prefer to use contraceptives.

With respect to access to media, the frequency of reading newspapers/magazines, frequency of listening to radio and frequency of watching TV showed significant association with use of modern contraceptive. Men who read newspapers/magazines at least once or below once a week were more likely to use male method than traditional/no method as compared to men who didn’t read newspapers/magazines at all. Similarly, men who listen to radio at least once a week were more likely to use male method than traditional/no method compared to men who didn’t listen to radio at all. In the same fashion, men who watch TV at least once or less than once a week were more likely to use partner method or male method than traditional/no method as compared to men who didn’t listen to radio or watch TV at all. As we compare to other findings [29, 30], these findings were consistent.

Unlike other studies done so far, wealth index [23, 31, 32], were not independently associated with modern contraceptive use in this study. The explanation can be from differences in study setting, sample size, study design and definitions.

## Conclusions

In this study, we considered multiple factors that contributed to modern contraceptive use among sexually active men in Ethiopia. Men with advanced age and urban residence were more likely to use modern contraceptives. In addition, men who were married and living with their spouse and men with educational level of secondary or higher were more likely to use modern contraceptives. The increasing number of ever born children was also positively associated with modern contraceptive use among sexually active men. Lastly, an exposure to sensitizing medias including newspaper, radio and TV increased the likelihood of modern contraceptive use among sexually active men in Ethiopia.

Thus, close monitoring and follow up of sexually active men with the above identified risk factors is highly recommended. Moreover, reproductive health programs aiming to increase uptake of modern contraceptives in this population of sexually active men in Ethiopia should consider the importance of men education and ensure access to media particularly, newspapers, radio and television.

## Data Availability

The data sets used and/or analyzed during the current study are available in the Ethiopian statistical agency and ministry of health.

## Abbreviations

CI: Confidence Interval
EDHS: Ethiopian Demographic Health Survey
AOR: Adjusted Odds Ratio
MEASURE DHS: Monitoring and Evaluation to Assess and Use Results Demographic and Health Surveys
